# Saponins Effect on Human Insulin Amyloid Aggregation

**DOI:** 10.3390/biom15010040

**Published:** 2024-12-31

**Authors:** Eleonora Mari, Silvia Vilasi, Paolo Moretti, Maria Rosalia Mangione, Giorgia Giorgini, Roberta Galeazzi, Maria Grazia Ortore

**Affiliations:** 1 Department of Life and Environmental Sciences, Marche Polytechnic University, I-60131 Ancona, Italy; 2 Institute of Biophysics, CNR, I-90146 Palermo, Italy

**Keywords:** amyloids, proteins, saponins, human insulin, small-angle X-ray scattering, circular dichroism, aggregation, molecular dynamics

## Abstract

The misfolding and amyloid aggregation of proteins have been attracting scientific interest for a few decades, due to their link with several diseases, particularly neurodegenerative diseases. Proteins can assemble and result in insoluble aggregates that, together with intermediate oligomeric species, modify the extracellular environment. Many efforts have been and are devoted to the search for cosolvents and cosolutes able to interfere with amyloid aggregation. In this work, we intensively study the effect of saponins, bioactive compounds, on human insulin aggregation. To monitor the kinetic of amyloid aggregation following secondary structure changes, we perform fluorescence and UV-Visible absorption spectroscopies, using Thioflavin T and Congo Red as amyloid specific probes, and Circular Dichroism. To study the overall structural features and size of aggregates, we perform Synchrotron Small-Angle X-ray Scattering and Dynamic Light Scattering experiments. The morphology of the aggregates was assessed by Atomic Force Microscopy. To deepen the understanding of the saponins interaction with insulin, a Molecular Dynamics investigation is performed, too. The reported data demonstrate that saponins interfere with the amyloid aggregation by inducing a strong inhibition on the formation of insulin fibrils, likely through specific interactions with insulin monomers. A dose-dependent effect is evident, and amyloid inhibition is already clear when saponins are just 0.01% *w*/*w* in solution. We suggest that saponins, which are natural metabolites present in a wide range of foods ranging from grains, pulses, and green leaves to sea stars and cucumbers, can be promising metabolites to inhibit human insulin aggregation. This basic research work can pave the way to further investigations concerning insulin amyloidosis, suggesting the use of saponins as amyloid inhibitors and/or stabilizing agents in solution.

## 1. Introduction

Saponins and their derivatives are glycosidic compounds playing several roles in food, agriculture, and pharmaceutical industries [1,2,3,4]. Although they have been suggested to have therapeutic potential as hypolipidemic [5], hypoglycemic [6], anti-asthmatic [7], antioxidant [8], anti-hypertensive [9], and anti-microbial activity [10], they show a few adverse effects due to their cytotoxicity. Their toxicity depends on their dosage, and it must be clearly declared that saponins vary much in their toxicity [11].

Saponins were recently supposed to play a role against neurodegenerative diseases, like Alzheimer’s Disease (AD) [12]. AD patients evidence the lacking of neuro-transmitters within the brain cells, due to the increase in acetylcholinesterase activity. As a consequence, acetylcholinesterase inhibitors (AchEIs) are believed to be among the best treatments of AD. A recent work compared the AchEI and apoptotic activities of fenugreek saponins against AD in vivo. Sprague Dawley rats were allocated in several experimental groups including untreated animals, animals supplemented with different doses of fenugreek saponin, and animals treated to induce AD. The results suggested that the ability of fenugreek saponin to inhibit AD might be attributed to antioxidant increase [12]. Recently, some of us proved that hop extracts, which are rich in saponins [13,14,15], can noticeably modify the amyloid aggregation pattern of human insulin and amyloid β-peptide [16] linked to AD, in agreement with previous results that tested the hop extracts’ ability to hinder amyloid β-peptide aggregation and cytotoxicity, and to enhance autophagy, promoting the clearance of misfolded and aggregated proteins in a human neuroblastoma SH-SY5Y cell line [17]. Since saponins are part of hop extracts, it appears interesting to investigate their proper contribution in inhibiting amyloid aggregation. Also, even more recently, a few papers suggested the promising role of saponins against neurodegenerative diseases [18,19]. Forty different types of saponins have been studied for their effects on AD, suggesting their potential to ameliorate AD by reducing amyloid β- peptide deposition, inhibiting τ phosphorylation, modulating oxidative stress, and reducing inflammation and antiapoptosis [19]. However, to our knowledge there is no systematic study on their effects on amyloid aggregation pattern in vitro, with the exceptions of two works dedicated to amyloid β peptide aggregation. In the first one, triterpenoid saponins from the cactus Polaskia chichipe were tested on amyloid β peptide [20], and in a second one, triterpene saponins from the cactus Stenocereus pruinosus were used on the same protein [21]. However, no biophysical characterization of the structure of aggregated species was reported in both the above-mentioned works, and a possible dose effect was not evidenced. On the other side, the biophysical characterization of protein species produced in the presence of saponins should improve the knowledge of the process and the understanding of the changes of molecular mechanisms leading to the formation of amyloid fibrils.

In order to get the picture of the potential inhibitory effect of saponins toward amyloidogenesis, we have planned a set of experiments considering a mix of saponins because they can represent a reproducible set already present in food and extracts. Also, we choose to investigate a protein whose amyloid aggregation is easily recordable and related to a pathological condition: human insulin. Insulin is considered a good model protein to study amyloid aggregation [22,23], and several investigations considered its amyloidogenesis at acidic pHs [24,25,26,27]. Also, it is known that during insulin production, the solution pH is acidic (between 2 and 4 [28]); hence, these chemical conditions facilitate aggregation, determining a reduction in functional activity and undesired immunological responses in patients [29]. Our investigation aims to study human insulin aggregation in conditions quite similar to those found in vivo, hence at the physiological pH. Although insulin aggregation at pH 7.4 follows a different pathway compared to acidic pH, involving distinct intermediates and the formation of morphologically diverse aggregates, the resulting fibrils still exhibit amyloid-like properties [26,30]. Insulin exists in solution in various oligomeric forms, including monomers, dimers, and hexamers, with their equilibrium depending on factors such as pH, ionic strength, protein concentration, and zinc ion levels, both in vitro and in vivo [26,30]. Under physiological conditions (37 °C and neutral pH), insulin amyloid formation is believed to originate from dimeric species, which are highly stabilized at this pH [31]. The propensity of insulin to aggregate even under these conditions makes it a suitable model system for studying pathological assembly phenomena under physiological conditions. Of note, although insulin amyloidosis is uncommon in mammals, several cases of amyloid deposits derived from insulin at injection sites have been linked to diabetes mellitus [32]. The human insulin fibrils structure deserves attention because its knowledge can be a starting point for the development of drugs that, binding to the fibril surface, can disrupt secondary nucleation, and very recently their atomic resolution structure was solved by magic angle spinning solid-state NMR spectroscopy [33]. However, in the above-mentioned study, insulin amyloid fibrils were obtained at quite a high temperature (60 °C) and at acidic pHs. On the other side, since insulin can aggregate at a physiological temperature after injection, we focus on its propensity to aggregate at 37 °C and physiological pH. We perform biophysical techniques able to monitor secondary, tertiary, and quaternary structure evolution, as well as Molecular Dynamics to understand the basis of insulin–saponin interactions. To this purpose, we choose as the reference compound a steroidal glicosidic saponin, Chichipenoside B, which already demonstrated its high anti-amilogenic activity [20]. In particular, Thioflavin T fluorescence and Congo Red absorption spectroscopy provide the β structure changes during the aggregation by using probes, while Circular Dichroism determines the amount of different secondary structure without the potential interference of probes. Dynamic Light Scattering obtains the average size of the final products of the aggregation phenomena in the presence and absence of saponins, while Synchrotron Small-Angle X-ray Scattering monitors the aggregation, obtaining the gyration radius of intermediate species. By using Atomic Force Microscopy, we assess the morphology of the insulin species formed in the presence and in the absence of saponins under amyloid aggregation conditions. Finally, by Molecular Dynamics, we pay attention to the interaction between Chichipenoside B and insulin molecules, suggesting the molecular basis of the inhibitory effect of saponins towards insulin amyloid aggregation.

## 2. Materials and Methods

### 2.1. Sample Preparation

Human Insulin recombinant was purchased from Sigma Aldrich (St. Louis, MO, USA), and it was used at a concentration of 0.5 mg/mL dissolved in phosphate buffer saline (50 mM) at pH 7.4, without further purification. Insulin solution was maintained at 4 °C for one hour, and the fibrillation process was induced at 37 °C with gentle agitation, according to the literature protocols [34]. Saponins were purchased from Sigma Aldrich as a mixture (CAS-No: 8047-15-2) and no composition details are reported. Samples were prepared with and without saponins, at various desired concentrations ranging from 0.1 mg/mL to 1 mg/mL, which are similar to those used for different saponins with Aβ peptide in the literature [20]. Saponins concentration ranges from 83 to 830 μM. Considering that human insulin concentration is 86 μM, the molar ratio between protein and saponins ranges from about 1 to 10.

### 2.2. UV-Visible Spectroscopy

We performed UV-Visible absorption spectra at regular time intervals, using Congo Red (CR) from Sigma Aldrich (St. Louis, MO, USA) as the amyloid specific probe, to monitor the kinetics of the amyloid aggregation of human insulin with thermostatic control at 37 °C, under gentle agitation. CR binding with β-structures induces a characteristic shifting of its absorption maximum in the UV/Vis spectrum, from about 500 nm to 540 nm. Because of its strong affinity for β-structures, distinctive of fibrillated proteins, CR dye has been employed as a recognizer for amyloidogenesis from many decades, and this approach has recently been considered valid for semi-quantitative analysis. To prepare CR solution, we first obtained a solution of ethanol (80% *w*/*w*), brought to saturation with NaCl. This solution was filtered and then saturated with the dye powder and filtered again with Millipore filters of pore size 0.2 μm to remove possible aggregates. We prepared each sample by adding 400 μL of the phosphate buffer at pH 7.4 and 50 μL of the selected protein sample with 50 μL of the Congo Red solution, in a quartz cuvette with 10 mm optical path. The amount of CR that we used for this kind of investigation was selected in previous works [35]. The ratio between absorption intensity at λ = 538 nm and at λ = 505 nm was calculated for each sample condition and reported as a function of time.

### 2.3. ThT Spectrofluorometric Measurements

The fluorescence emission of Thioflavin T (ThT, Sigma-Aldrich) was monitored in time using a 96-well microplate with thermostatic control at 37 °C. We selected as excitation and emission wavelengths 450 and 485 nm, respectively. All measurements were performed in triplicate. Human insulin (Sigma-Aldrich) was dissolved in a phosphate saline buffer 50 mM, at pH 7.4 at 0.5 mg/mL, as in the other experiments. In order to monitor the insulin amyloid aggregation kinetics, the protein samples in the presence of ThT were incubated at 37 °C under agitation with teflon beads, for at least 40 h. Although these beads do not achieve shear rates as high as those produced by magnetic stirring, they do ensure the formation of on-pathway amyloid seeds, which are crucial for triggering subsequent fibrillogenesis [36].

The protein solution was investigated in the presence and in the absence of increasing amounts of saponins.

### 2.4. Circular Dichroism

Circular Dichroism (CD) measurements were recorded after 48 h of aggregation kinetics at 37 °C and were performed at room temperature by using a JASCO J-810 spectrometer (JASCO Corporation, Tokyo, Japan) equipped with a temperature control unit. The investigated protein solutions were exactly the same monitored by ThT fluorescence. Quartz cells with 0.5 mm path lengths were used for registering CD spectra in the far UV region. Each CD spectrum was corrected for the baseline by subtracting the spectral contribution of the saponins alone. The estimation of the secondary structure was carried out using the BestSel web server [37].

### 2.5. Dynamic Light Scattering

Dynamic Light Scattering (DLS) measurements were performed on a Malvern Zetasizer Pro instrument (Malvern Panalytical, Malvern, UK) configured in back-scattering geometry (scattering angle, θ = 173°), using an incident wavelength λ = 633 nm, produced by a He–Ne laser. The main advantage of this detection geometry is that it is less sensitive to multiple scattering effects. Human insulin solutions at 0.5 mg/mL were loaded into standard quartz cuvettes (10 mm optical path) and kept at 25 °C for the measurements after 5 h of the aggregation kinetics at 37 °C under gentle agitation, which was performed with the same method we used for UV/Vis spectroscopy experiments. Each experiment was performed with three replicas. The intensity autocorrelation function $g_{2}\left(\tau \right)-1$ was computed automatically by the instrument, where τ is the correlation constant. To obtain the size distribution, the measured autocorrelation functions were analyzed using the CONTIN algorithm [38].

### 2.6. Small-Angle X-Ray Scattering

Small-Angle X-ray Scattering (SAXS) data were collected at the Austrian beamline of Elettra Synchrotron in Trieste, Italy [39]. Kinetics in the presence and in the absence of saponins were monitored in time using a microplate with thermostatic control at 37 °C under agitation. Protein solutions (at concentration c = 0.5 mg/mL) and buffers were measured in the same conditions concerning temperature and exposure time. The data collected were corrected for sample transmission and primary beam fluctuations. We carefully checked each set of scattering patterns and performed the average after a positive control over radiation damage. We did not observe radiation damage on the samples presented in this study. Single scattering patterns were derived from the average of all images in each sample; the respective medium backgrounds, treated in the same way, were subtracted from the average of all images. The sample was collected and then injected by the µ-Drop Sample Changer developed in the Austrian beamline [40]. Each measurement was performed by averaging the results of 3 injections of 20 μL of the same sample. For each injection, we obtained 18 images, each one resulting from an exposure time of 10 s. To minimize the radiation damage effects in the protein sample, each SAXS acquisition was followed by 3 s of dead time. Buffer measurements were always performed before and after sample measurements. Protein solutions and buffers were measured under the same conditions regarding temperature and exposure time.

### 2.7. Atomic Force Microscopy

Atomic Force Microscopy (AFM) measurements were acquired on an AIST-NT scanning probe microscope (Horiba Scientific, Kyoto, Japan) in non-contact mode. A silicon pyramid tip with a radius of 8 nm was used to perform the measurements. Samples were prepared after reaching the fibrillation end point after 5 h of the aggregation kinetics at 37 °C, which was performed in the same way we carried out the UV/Vis spectroscopy experiments and diluted from the original concentration by a factor of 1:10. This factor was optimized to avoid the deposition of aggregates and artifacts on the mica surface. A total of 10 μL of the diluted solution was deposited on the freshly cleaved mica and allowed to incubate for 10 min. Next, the mica surface was rinsed with milli-Q water and dried with nitrogen flushing. All images were acquired with a scan rate of 1 Hz and a resolution of 512 × 512 pixels. Image analysis was performed with Gwyddion software 2.67 [41]. For each sample, at least 5 images were acquired and analyzed.

### 2.8. Molecular Dynamics

Insulin 3D structure was retrieved from PDB data Bank (code 1ev3 [42]) and prepared for the subsequent computational simulations in its monomeric form, adding hydrogen and charges using AM1-BCC methods (within CHIMERA-1.18 software [43]) followed by minimization using the GB/SA implicit solvent method for water [44]. Chichipenoside B, a terpenoid saponin steroidal glycoside derived from Chichigenin, was chosen because Fujihara et al. observed the amyloid β aggregation inhibitory activity of ten triterpene saponins of natural origin [20,45], and among these compounds, we chose one of the most active, Chichipenoside B, to test its inhibitory power against insulin aggregation to shed light on its effect at the molecular level. The Chichipenoside B structure as a saponin representative compound was built and modeled using GaussView/G09 software and its charges were calculated at the B3LYP/6-311G(d,p) level of theory [46]. Then, docking calculations were carried out to evaluate the saponin–protein binding affinity, performing blind docking all over the insulin surface by MGLTols/Autodock 4.2 (GA algorithm for cluster generations, LA-GA for subsequent analysis) [47]. The docking parameters were set to default values except for the number of GA runs (100), the energy evaluations (25,000,000), the maximum number of top individuals that automatically survive (0.1), and the step size for translation (0.2 Å). The best clusters underwent a focused docking protocol in order to refine the binding energy and for pose centering the grid maps on the ligand. The clusters’ analysis showed the prevalence of one docking pose configuration that was then used for the subsequent Molecular Dynamics (MD) simulations to assess the stability of the saponin–insulin association.

The MD of the protein and saponin system was carried out in periodic boundary conditions (PBCs) using explicit TIP3P water models. The simulated system consists of four unities of the insulin–saponin complexes as obtained from the previous docking calculations, put randomly within the cubic water TIP3P box. Besides this system, we also simulated a system containing two molecules of Chichigenin B and insulin in dimer association (1ev3 pdb code), and finally a system containing 4 molecules of saponin and 4 insulin monomers. The composition of the system was chosen to achieve the smallest possible setup (in a periodic water box) that allows for reasonable computation times and scalability on HPC systems, while minimizing potential bump-checks, overlaps, or repulsive interactions between molecules. This ensures that the molecules can move freely within the simulation box. Additionally, the equimolar ratio was selected to statistically evaluate the intermolecular interactions between insulin molecules and saponins, providing insight into which interactions may be energetically favored. The GROMACS 2020.6 suite of programs was used to perform all simulations using the CHARMM36 force field parameter sets, and the Chichipenoside B’s charges and parameters previously calculated by DFT methods by using CHARMM-GUI Solution Builder. After minimization, the system underwent an NVT equilibration of 5 ns followed by the 200 ns production phase carried out on the NPT ensemble at 1 atm and 310 K (37 °C). The Parrinello–Rahman barostat was used to keep the pressure constant isotropically and the Nosè–Hoover thermostat [48], setting the time constant for coupling to 0.5 ps. Electrostatic interactions were taken into account by the Particle Mesh Ewald algorithm, with a 1.2 nm distance for the Coulomb cut-off, to calculate the long-range electrostatic interactions in the periodic system; the LINCS algorithm was used to freeze the H-bond stretching during the simulation. The MD trajectory convergence was monitored by the rmsd graph which reached a plateau with a ΔRMSD < 0.1. The distance between the Chichigenin B molecules and insulin along the MD trajectory was calculated using the mindist tool in GROMACS that calculates the minimum distance between two groups of atoms, which can represent two molecules or parts of molecules. It determines the shortest distance between any pair of atoms from the two groups at each frame of the simulation trajectory. In our case, we considered the center of mass of each molecule.

## 3. Results

### 3.1. Secondary Structure

To investigate the insulin amyloid formation, we incubated insulin at 37 °C with mechanical agitation. This setup promotes nucleation, a key step in the “on-pathway” amyloid fibrillation process. In fact, stirring or shaking has been shown to accelerate polymerization by breaking apart large aggregates and increasing the frequency of collisions between reactive molecules and fiber ends. Furthermore, higher temperatures improve hydrophobic interactions, lowering energy barriers for β-sheet amyloid formation, which in turn can serve as seeds for a subsequent on-path structure [49]. UV-Vis Spectroscopy in the presence of Congo Red (CR) and Fluorescence Spectroscopy in the presence of Thioflavin T (ThT) were used as complementary tools to evaluate secondary structure changes during insulin aggregation. Furthermore, we performed CD experiments on the same set of samples in order to demonstrate that the two probes (CR and ThT) did not affect the kinetic pattern and saponins effect on protein aggregation into amyloid fibrils.

**Figure 1:**
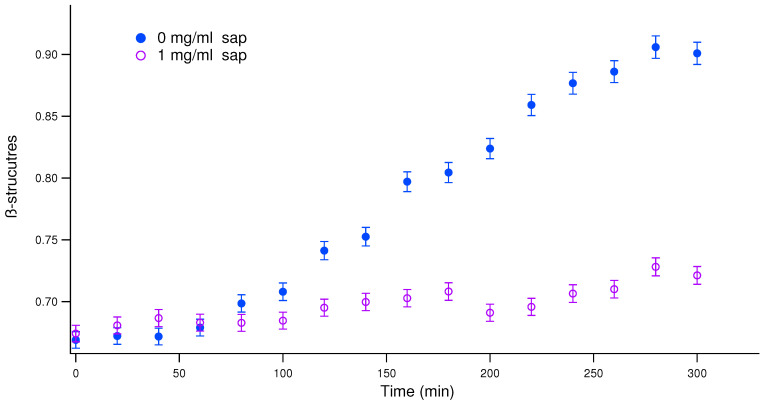
UV/Vis spectroscopy results of the effects of saponins on human insulin aggregation. To monitor the relative amount of β-sheet structures in solution, the ratio between the intensity of the absorption peak due to Congo Red (CR) bound to fibrils and the one due to CR free in solution was calculated as a function of time. β-sheet structures as a function of time during fibrillation: blue markers correspond to human insulin in solution in the absence of saponins, and purple markers correspond to human insulin in solution in the presence of 1 mg/mL of saponins. Error bars are estimated on the average of several replicas of the same experiment.

In Figure 1, the relative quantity of the β-structures was obtained from the ratio of the absorption intensity at λ = 538 nm and the one at λ = 505 nm as in previous works [35,50]. The presence of 1 mg/mL of saponins in solution clearly inhibits insulin aggregation. Also, the results from increasing the saponins concentration reported in Figure S1 in Supplemental Materials indicate a dose-dependent effect.

**Figure 2:**
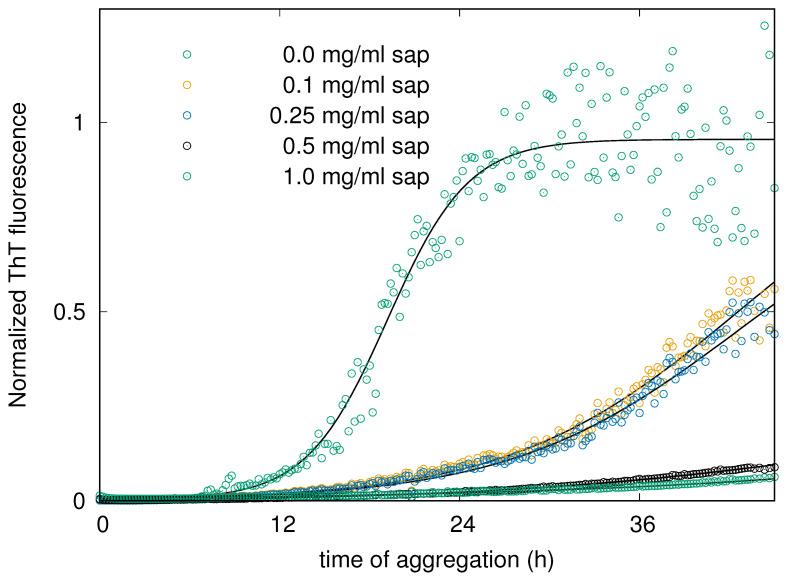
In situ real-time ThT fluorescence assay for monitoring the aggregation kinetics of insulin incubated at 37 °C under agitation in the absence and in the presence of increasing concentrations of saponins as indicated in the legend. Continuous lines are the theoretical fitting curves obtained by the analysis that provides the halftime featuring each kinetic pattern.

The ThT results, reported in Figure 2, provide the amount of β structure during human insulin aggregation at saponins concentrations ranging from 0 to 1 mg/mL. Even these data confirm that saponins interfere by a dose-dependent mechanism with amyloidogenesis. The sigmoid growth of the signal can be easily appreciated and consequently quantitatively evaluated by obtaining the time needed to reach the first half of the process. The halftime of the aggregation process is considered as a measure of the mutational effects on nucleation, as well as an indicator for the overall nucleation rate when both primary and secondary nucleation reactions are present [51,52]. Halftimes resulting from the theoretical fit reported in Figure 2 are shown in Table 1. The human insulin aggregation at pH 7.4 and at physiological temperature (37 °C) performed for fluorescence spectroscopy presents a kinetic whose halftime is about 20 h. We underline that this value is highly affected by the environment and by the aggregation scenario, as it happens for each induced amyloid pattern [34,53]. That is why we greater appreciate the differences in halftimes obtained in various solvent conditions, and we pay less attention to the absolute time values. Just a very low amount of saponins, 0.1 mg/mL (hence 0.01% *w*/*w*), determines the double of the halftimes (see Table 1). Increasing again the saponins content in solution, the halftimes increase again, and no real sigmoidal curve appears (see the light green circles in Figure 2).

**Figure 3:**
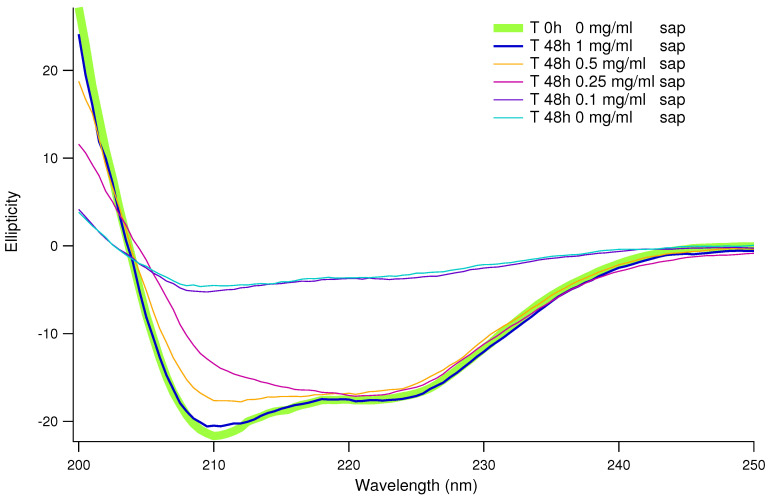
Circular Dichroism (CD) curves of human insulin recorded after 48 h of aggregation kinetics at 37 °C. The curves are related to the human insulin solution in the presence of increasing amounts of saponins as indicated in the legend.

**Figure 4:**
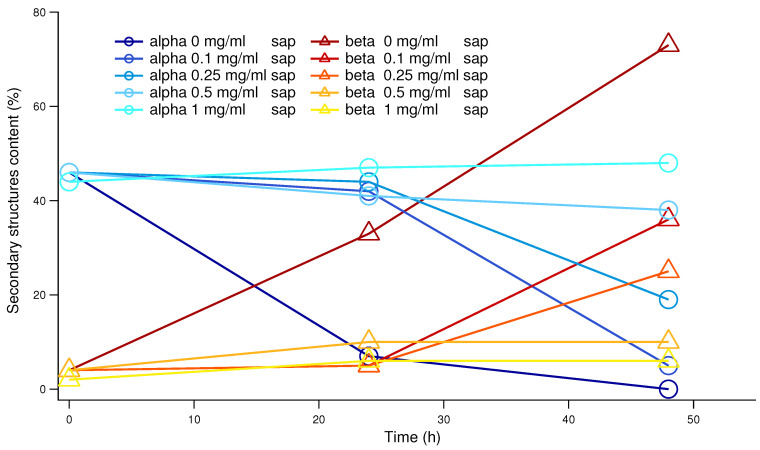
Percentages of α and β structure, as in the legend, provided by CD data analysis. Blue and cyan shades refer to α-helices, which dramatically decrease in the absence of saponins. On the contrary, in the same conditions, β structures arise, in shades from red to yellow.

To confirm that the saponins effect in inhibiting human insulin amyloid formation is not affected by the presence of CR and/or ThT, we performed a set of CD experiments in the absence of probes. Human insulin aggregation was induced at physiological pH and temperature, and under exactly the same conditions adopted for the ThT fluorescence experiments (but in the absence of ThT and teflon beads). The CD spectra resulting from the average of at least 3 replicas are shown in Figure 3. The green thick continuous line reports the human insulin CD fingerprint at a physiological pH, which shows two characteristic minima, at 211 nm and 222 nm, indicative of a prevalence of an α-helical structure as reported in the literature [26,54]. This spectrum remained unchanged when saponins were added to the insulin solution prior to inducing amyloid aggregation through mechanical agitation and increasing temperature (see Figure S7 in Supplemental Materials). This suggests that no significant changes in the protein secondary structure were detectable upon saponin binding. Taking into account the previous ThT fluorescence results (see Figure 2), all the insulin stocks including different amounts of saponins were collected after 24 and 48 h from the onset of induced aggregation. It can be easily appreciated that without saponins (cyan line in Figure 3), after 48 h insulin, the CD spectrum was completely changed. While a similar behavior can be traced in the sample at low saponin content (0.1 mg/mL, blue curve in Figure 3), when the saponin concentration doubled at 0.25 mg/mL, the CD spectra were nearer to the native one characterized by approximately ≃50% of α-helix content and a minimal β-sheet structure of around ≃4%. Increasing again the saponins content, we observed that the CD spectra were getting closer to the native one. In particular, at 1 mg/mL of saponins in solution, after 48 h, the CD spectrum practically overlapped the native one. To better understand CD spectra evolutions and the dose dependence of the saponins effect, CD data were theoretically fitted in order to estimate the content in secondary structures. In Figure 4, the α-helix content and the β-sheet content are reported as a function of time for the saponins concentrations ranging from 0 to 1.0 mg/mL. With or without saponins, human insulin at the beginning of the aggregation process shows ≃50% α-helix content and a low percentage of the β-sheet structure. Significant α-helix to β-sheet transition was observed already after 24 h, in the absence of saponins. After 48 h, almost ≃80% of the secondary structures correspond to β-sheets, for human insulin in aggregation without saponins. On the other side, when 1 mg/mL of saponins are dissolved in solution, the trend of secondary structures (cyan circles and yellow triangles in Figure 4) is practically constant until 48 h. These data strongly reinforce the previous UV-Vis and fluorescence results, confirming the inhibitory effect of saponins and its dose-dependent impact.

### 3.2. Three-Dimensional Structure

The protein aggregation pattern leading to amyloid fibrils is commonly and mainly characterized by the evolution of secondary structures and, in particular, of β-sheets. However, the overall structure of the amyloid aggregates, which can resemble fibrils, protofibrils, and oligomers, is really important for cytotoxicity [55,56], and consequently is something that deserves investigation.

**Figure 5:**
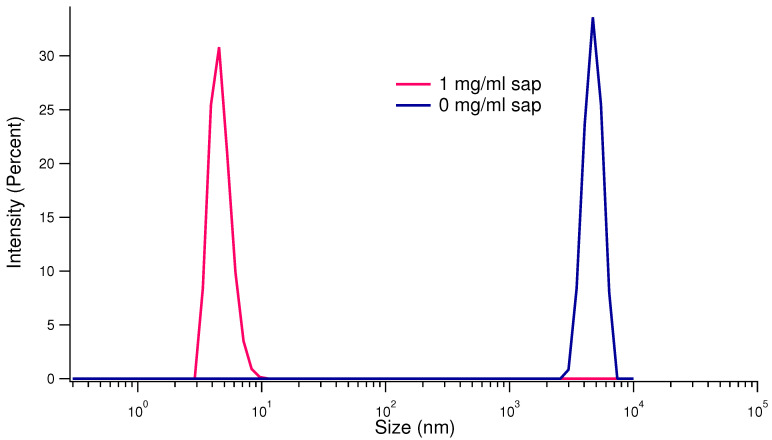
DLS results: particle size distribution of human insulin at 0.5 mg/mL, after 5 h of kinetics of aggregation in the presence (pink line) and in the absence (blue line) of saponins.

Firstly, we performed DLS experiments on the final states of the kinetics monitored by UV-Vis absorption spectroscopy, conducted in the absence and in presence of saponins. We choose the final states as the time steps when we already observed by UV-Vis absorption spectroscopy the saturation of the signal due to protein aggregation. Autocorrelation functions were analyzed by Malvern Zetasizer 3.30 software and provided a well-defined size distribution for each set of experiments. The results are shown in Figure 5: the size distribution curves refer to insulin at the supposed end of the agitation-induced aggregation processes. It is evident that the blue curve, referred to the sample without saponins, shows the presence of big aggregates, whose average size is in the range of μm. On the other side, the pink curve, which refers to the final aggregation product obtained in the presence of 1 mg/mL saponins, corresponds to native insulin average size. Note that the particle size distribution obtained by DLS data is obtained by Stokes–Einstein approximation, hence according to the hypothesis of the presence of almost spherical nanoparticles. This approximation does not completely agree neither with insulin native conformation nor with amyloid products. However, the size estimated difference of the aggregation products is so relevant that we can neglect the bareness of the approximation.

**Figure 6:**
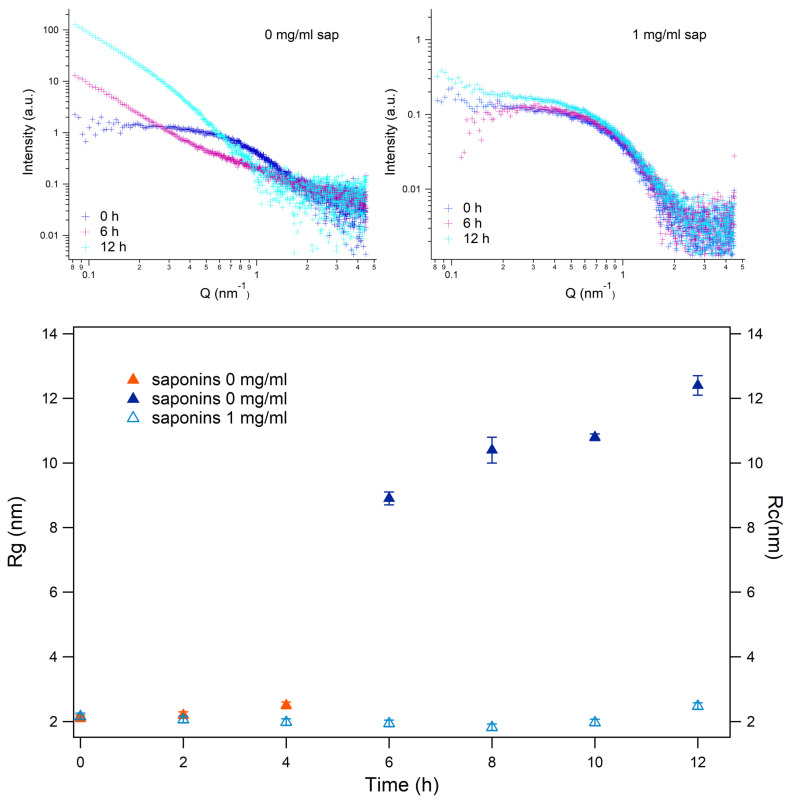
SAXS results. Upper panels: SAXS spectra relative to human insulin (c = 0.5 mg/mL, pH 7.4) aggregation patterns at the beginning (0 h, in the legend, blue points), and after 6 (purple points) and 12 h (cyan points). On the left side, data concern insulin dissolved without saponins, and on the right side are data collected in the presence of 1 mg/mL saponins as shown in the legend. Bottom figure: structural parameters obtained by Guinier (Rg) and Guinier rod-like (Rc) analysis as a function of aggregation time. The empty cyan triangles represent the parameters achieved (Rg) for the curves obtained for the kinetics in the presence of saponins, while the orange (Rg) and the blue (Rc) triangles represent the parameters achieved for the kinetics in the absence of saponins.

To improve the characterization of the species resulting from the aggregation process, we took advantage of synchrotron SAXS, a technique which provides structural information on the different species, at different resolutions, according to the data analysis approach [57,58,59,60]. We performed SAXS experiments along the aggregation pattern, which are summarized in the top panels of Figure 6. The saponins presence in solution at a concentration of 1 mg/mL, even in this experiment, is able to noticeably interfere with insulin aggregation, inhibiting almost completely the amyloidogenesis. At fixed temporal intervals of two hours, SAXS curves were collected for each condition (with and without saponins). SAXS data were analyzed according to a model-free approach, in order not to overestimate the intrinsic structural content of the experimental results. Hence, a Guinier approximation $I\left(Q\right)=I_{0}e^{-\frac{R_{g}^{2}Q^{2}}{3}}$ was used if the experimental data could be successfully fitted, confirming the presence of species with spherical symmetry whose gyration radius is $R_{g}$. On the other side, a Guinier rod-like approximation $I\left(Q\right)=\frac{A}{Q}e^{-\frac{R_{c}^{2}Q^{2}}{2}}$ was adopted at higher aggregation times, where $R_{c}$ is the cross-sectional radius of gyration, which is related the overall size of the cross section of the particle. Note that Guinier rod-like approximation was used when simple Guinier approximation failed in fitting the experimental data. At the beginning of the aggregation process, the estimated gyration radius was $R_{g}≃2$ nm both in the presence and in the absence of saponins, in agreement with the insulin dimer size previously reported [61] as shown in Figure 6, bottom panel. In the absence of saponins, the Guinier approximation of spherical particles dispersed in solution failed after 6 h of kinetics, while a Guinier rod-like approach could provide a $R_{c}≃9$ nm (blue triangles in Figure 6, bottom panel). The analysis of SAXS curves corresponding to human insulin during the aggregation process induced in the absence of saponins shows that the cross section radius slightly increased until 12 h from the beginning of the phenomenon, reaching $R_{c}≃12\mathrm{nm}$. When 1 mg/mL saponins were added to the human insulin solutions, in contrast, the gyration radius was maintained at about the starting values (cyan triangles in Figure 6, bottom panel), confirming their ability to interfere with amyloid aggregation. In particular, SAXS results demonstrate that saponins were able not just to deny the conversion of α-helix structures to β-sheets, as shown by UV-Vis and Fluorescence Spectroscopy but even to prevent insulin from aggregation, as the gyration radius after 12 h just slightly increased (see cyan triangles in Figure 6).

**Figure 7:**
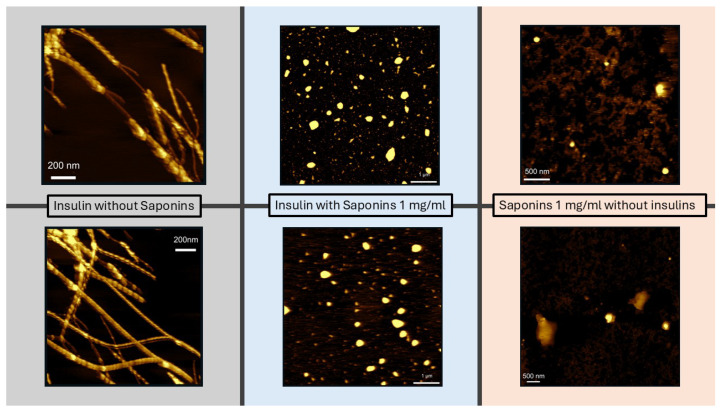
Atomic Force Microscopy images. Top and bottom images are two replicates of the same sample. Grey panel: insulin without saponin. Light blue panel: insulin with saponins (1 mg/mL). Powder pink panel: saponin without insulin (1 mg/mL).

To more deeply investigate the morphology of the human insulin aggregation products, we performed Atomic Force Microscopy (AFM) imaging of the batches at the endpoint of the aggregation kinetics, in the presence and in the absence of saponins in solution. We performed AFM experiments on insulin without saponins, and insulin with saponins, at the end of the aggregation process (24 h), and on saponins solutions without insulin, too. The height of the fibers reported in the images in the gray panel of Figure 7, which correspond to human insulin at the end of the aggregation phenomenon, is within a few nanometers, in agreement with previous works [35,62,63]. AFM images are shown in Figure 7: while fibrillar aggregates are clearly evident in the gray panel, as the result of the human insulin aggregation process, in the light cyan panel, corresponding to insulin aggregated in the presence of saponins, no fibril appears. On the other side, several aggregates whose shape is similar to the spheres appear. In order to check if these aggregates could be associated to saponins or to proteins, further AFM experiments were carried out on the saponins solutions at the same concentration adopted to inhibit amyloidogenesis. In the pink powder panel of Figure 7, almost spherical aggregates can be evidenced, very similar to those detected in the cyan panel. Saponins can self-assemble to form spherical micelles above a critical micelle concentration (cmc), whose value for Quillaja Saponin ranges between 0.5 and 0.8 mg/mL [64]; hence, the deposition of the saponins solutions on the mica surface can induce a self-assembly which does not appear in solution.

To quantitatively compare the aggregates visualized in the central panel of Figure 7 (insulin with saponins) to the ones reported on the right (saponins alone), we reported the heights of the aggregates as the average of 20 height measurements for each sample (Figure S3 in Supplemental Materials). The average height of the aggregates in the samples of insulin with saponins was slightly lower than that of the samples with saponins alone. This result can be related to the hypothesis that protein particles bind to saponins molecules in solution, lowering their absolute concentration as free molecules dispersed in the buffer, and determining the formation of micelles of a smaller size. However, all the above-mentioned biophysical techniques cannot prove the hypothesis on saponins binding to insulin.

### 3.3. Molecular Findings

Molecular Dynamics simulations were performed in order to understand possible direct interactions between human insulin and saponins thus unveiling the molecular mechanisms underlying their amyloid inhibitory action. Since human insulin aggregation according to our protocols is not proved to be determined by protein misfolding, we supposed that saponins can somehow bind to insulin, inhibiting their aggregation by stabilizing their structure and energetic landscape.

**Figure 8:**
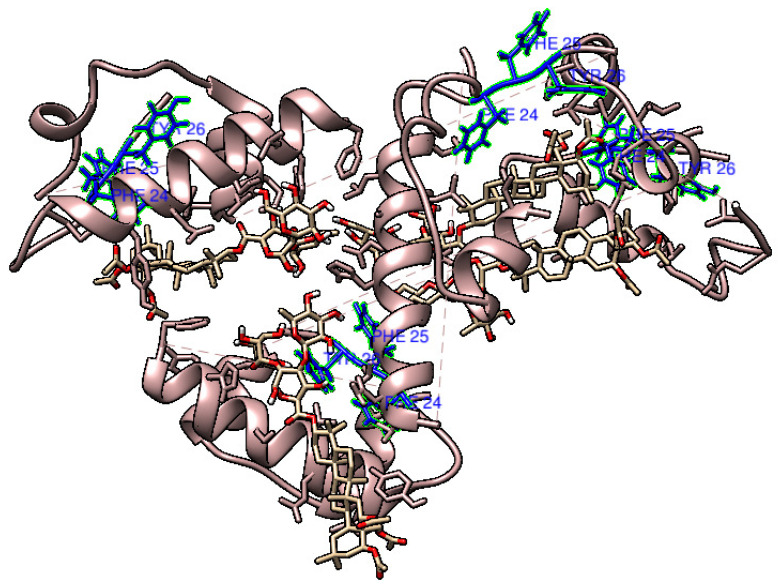
Chichipenoside B (in tube representation) and insulin (ribbons) molecular organization at the final steady state of the 200 ns MD simulation.

**Figure 9:**
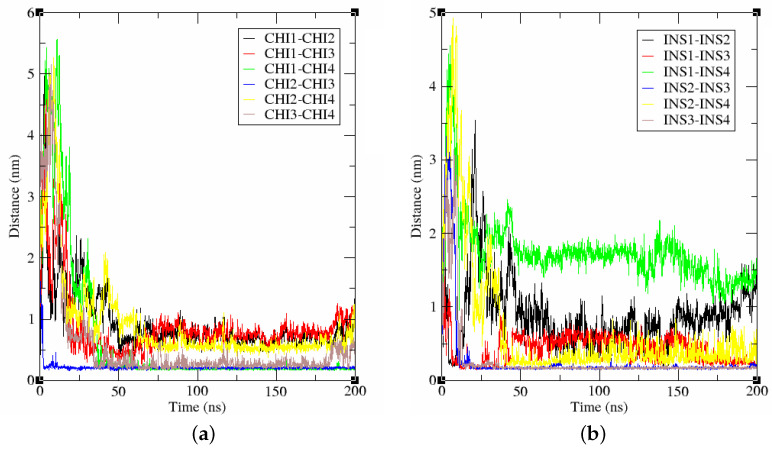
Intermolecular distances between the four Chichipenoside B molecules (**a**) and insulin (**b**) along the 200 ns MD simulation.

Along the simulation trajectory, we can observe increasing intermolecular interactions between the four protein–ligand unities, that came closer along the trajectory till reaching the equilibrium state (see Figure 8 and Figure 9). As can be seen in Figure 8, we can assist in an intercalation of the Chichipenoside B molecules within the insulin–protein unities. We calculated the intermolecular minimum distances (center of mass) between Chichipenoside B unities (Figure 9a) and insulin proteins (Figure 9b).

**Figure 10:**
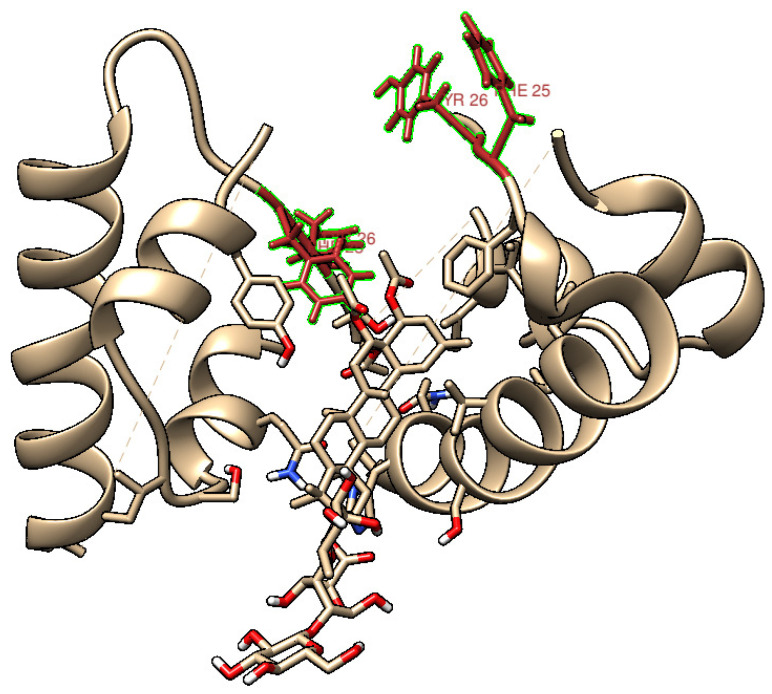
Visualization obtained from the 200 ns MD simulation of Chichipenoside B and human insulin, evidencing the residues playing a fundamental role in dimerization.

More specifically, MD simulations indicate that Chichipenoside B may specifically interact with residues Phe24, Phe25, and Tyr26 (see Figure 10), which play a critical role in dimer formation [65]. The distance between these specific residues and the Chichipenoside B are reported in Figure S5 in Supplemental Materials. It is easy to observe that, despite fluctuating throughout the time of the dynamic evolution, the interaction is maintained, in agreement with the hypothesis that saponins strongly interfere with monomers’ association into dimers.

Hence, we can suggest that the stabilization of the dimerization interface may hinder protein dimerization itself and, as a result, inhibit amyloid aggregation in human insulin, confirming the previous hypothesis [26]. No protein misfolding has been observed during the simulations, in agreement with the experimental findings.

## 4. Discussion

Human insulin aggregation pattern leading to amyloid fibrils has been here investigated in conditions quite similar to those in vivo, i.e., at physiological pH and temperature. In light of the recent findings on the insulin fibrils structure [33] and on insulin-derived amyloidosis [66], it appears important to look for the potential inhibitors of its aggregation. In fact, while insulin treatment has become increasingly essential for patients living with diabetes over the past few decades, insulin-derived amyloidosis (AIns) is a skin complication of insulin therapy. Although AIns was discovered about 40 years ago, no pharmacological therapy is available. The main concern is that AIns causes poor glycemic control and leads to increased insulin dosage in patients with diabetes due to impaired insulin absorption. Nowadays, most common therapies are changing the injection site and surgery, but with our study, we propose the possibility to add a natural cosolute, saponins, to meaningfully decrease the insulin aggregation propensity.

Our biophysical experiments demonstrate the ability of saponins to inhibit insulin amyloid aggregation monitoring secondary structures changes, by ThT fluorescence and CR absorption spectroscopy, and by CD spectra analysis. Also, DLS and SAXS demonstrate that saponins do not induce the formation of other kind of aggregates, and this result is confirmed by AFM images. In order to provide a potential molecular explanation to this effect, MD was performed on insulin and a specific saponin, demonstrating a direct interaction that could stabilize certain insulin conformations. The dissociation of an insulin dimer to two monomers is an important life process [67] because although the monomer is the biologically active form of the hormone, it is stored in the pancreas in the hexameric form. The latter dissociates to dimers and the dimers, in turn, to monomers to maintain the endogenous delivery of the hormone. Previous MD work calculated the free energy landscape and determined the minimum free energy pathway of dissociation, providing a pathway involving multiple minima and multiple barriers [67]. It has been shown that the insulin dimer, the predominant association state at neutral pH, is the species responsible for triggering amyloid formation at neutral pH by transitioning into a nucleus through an unidentified intermediate state [26,31]. Molecular Dynamics (MD) results suggest that saponins may directly interact with the insulin monomer residues Phe24, Phe25, and Tyr26, which, in the native dimer structure, are located at the interface between the subunits [65]. Our hypothesis is that this interaction could stabilize the monomers, shifting the equilibrium between monomers and dimers in favor of the monomeric form. This would prevent the formation of those dimeric species, which serve as critical seeds for triggering the fibrillation pathway. By inhibiting the formation of these key seeds, the amyloid aggregation process in insulin would be halted, effectively blocking the development of amyloid fibrils. In conclusion, our results demonstrate that, in the absence of saponins, insulin aggregation at physiological pH and temperature, triggered by mechanical agitation, induces a structural transition from the native α-helical conformation to a β-sheet amyloid structure. This process, which likely involves dimers as transitional intermediates, is prevented by saponins. Saponins preferentially bind to insulin monomers at the dimerization interface, restricting their conformational flexibility and thereby inhibiting aggregation. Although our hypothesis requires more sophisticated structural methodologies to be experimentally verified, and we plan to pursue this, our results on the total inhibition of insulin amyloid formation exerted by saponins provide an important starting point for imagining strategies to prevent amyloidosis at the injection site. We consider that the addition of saponins to insulin injected for therapies, as well as saponins used as active principles for topic application, could be a promising possibility for AIns disease.

## Figures and Tables

**Table 1:** Halftimes of human insulin aggregation process at pH 7.4 and 37 °C, in the presence of different saponins concentrations.

Saponins Concentration (mg/mL)	0	0.1	0.25	0.5	1.0
Halftime (h)	19.2 ± 0.1	42.6 ± 0.7	42.6 ± 0.7	52 ± 1	110 ± 80

## Data Availability

The raw data supporting the conclusions of this article will be made available by the authors on request.

## Supplementary Materials

The following supporting information can be downloaded at: https://www.mdpi.com/article/10.3390/biom15010040/s1, Figure S1: UV/Vis spectroscopy results on the effects of saponins on human insulin aggregation in the absence and in the presence of increasing concentration of saponins, as indicated in the legend. Figure S2: Average height of aggregates derived from sample of insulin with saponins and saponins alone. Figure S3: Small Angle X-ray Scattering (SAXS) curves of human insulin at the beginning of the aggregation. Figure S4: Experimental SAXS curves of human insulin at the end of the kinetics of aggregation. Figure S5: Intermolecular distances between the four Chichigenin B molecules and Phe24, Phe25 and Tyr 26 of insulin along the 200 ns MD simulation. Figure S6: 1:1 Chichigenin B/insulin–left top view, right side view. Steady state structure after 100 ns MD simulation in water solution. Figure S7: CD spectra of human insulin in presence and in absence of 1 mg/mL saponins, together with saponins solution, as in the legend. Insulin CD spectrum compared with the one obtained from the difference between the insulin-saponins sample and the saponins-alone sample, attributed to the structure of insulin bound to saponins, are very similar.

## Abbreviations

The following abbreviations are used in this manuscript:

AD	Alzheimer’s Disease
AchEI	Acetylcholinesterase inhibitors
ThT	Thioflavin T
CR	Congo Red
CD	Circular Dichroism
SAXS	Small-Angle X-ray Scattering
DLS	Dynamic Light Scattering
AFM	Atomic Force Microscopy
MD	Molecular Dynamics
AIns	Insulin-derived amyloidosis
