# Supplementary material for: Saponins Effect on Human Insulin Amyloid Aggregation
Source: Biomolecules. 2024 Dec 31;15(1):40. doi: 10.3390/biom15010040 (PMC11762657; doi:10.3390/biom15010040)
Supplement: Supplementary file 1 [file biomolecules-15-00040-s001.zip › biomolecules-3367807-supplementary.pdf]

SUPPLEMENTARY MATERIALS

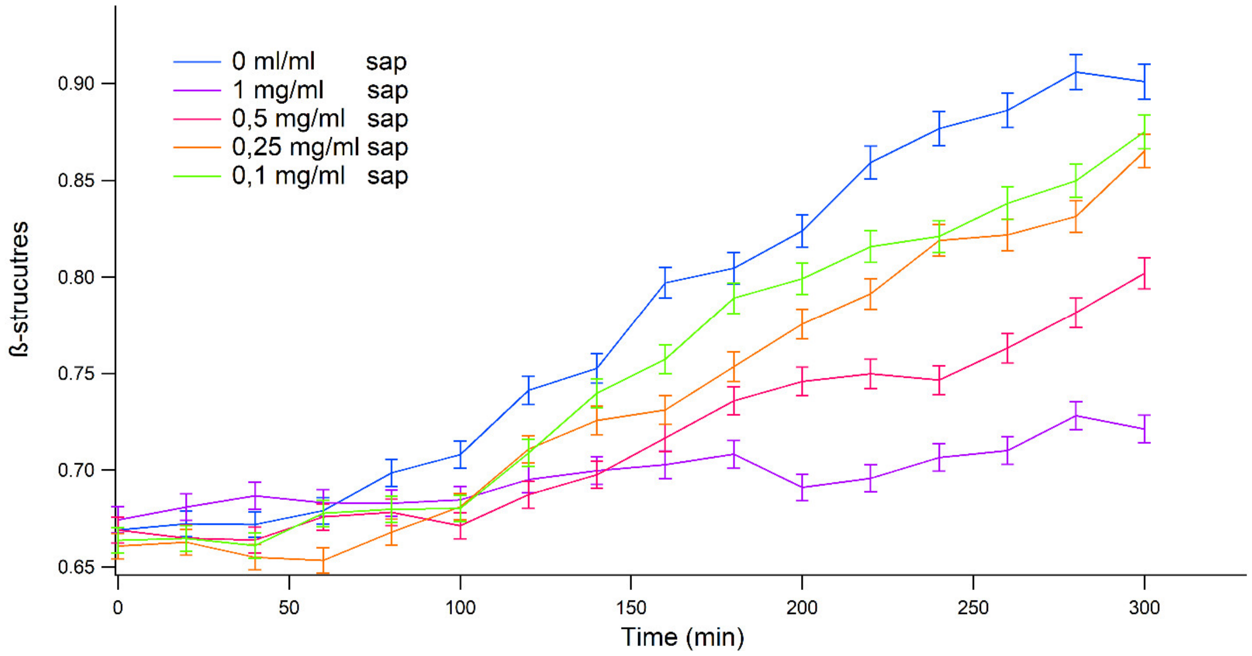

Figure S1. UV/Vis spectroscopy results on the effects of saponins on human insulin aggregation in the absence and in the presence of increasing concentration of saponins, as indicated in the legend. To monitor the relative amount of β-sheet structures in solution, the ratio between the intensity of the absorption peak due to Congo Red (CR) bound to fibrils and the one due to CR free in solution was calculated as a function of time. Error bars are estimated on the average of several replicas of the same experiment.

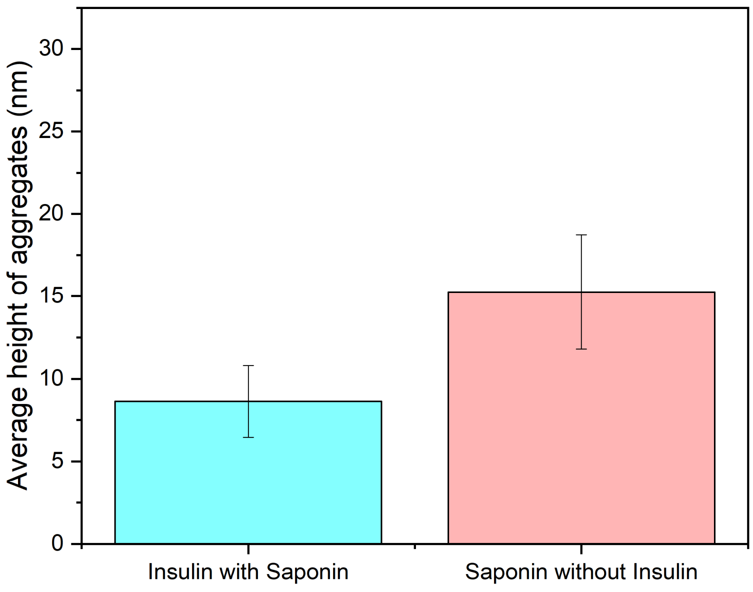

Figure S2. Average height of aggregates derived from sample of insulin with saponins and saponins alone.

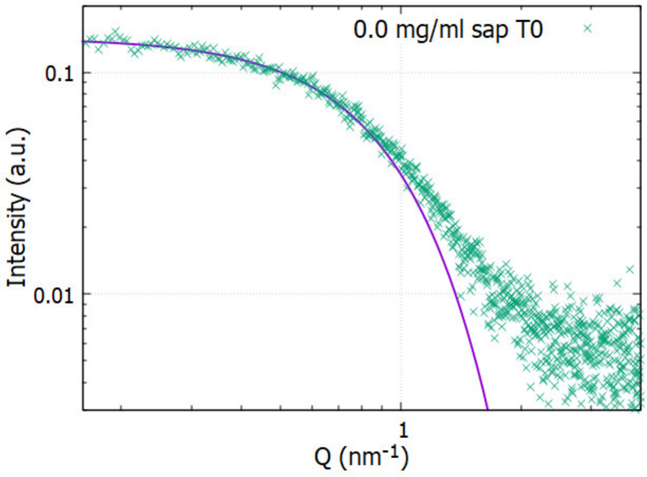

Figure S3. Small Angle X-ray Scattering (SAXS) curves of human insulin at the beginning of the aggregation.

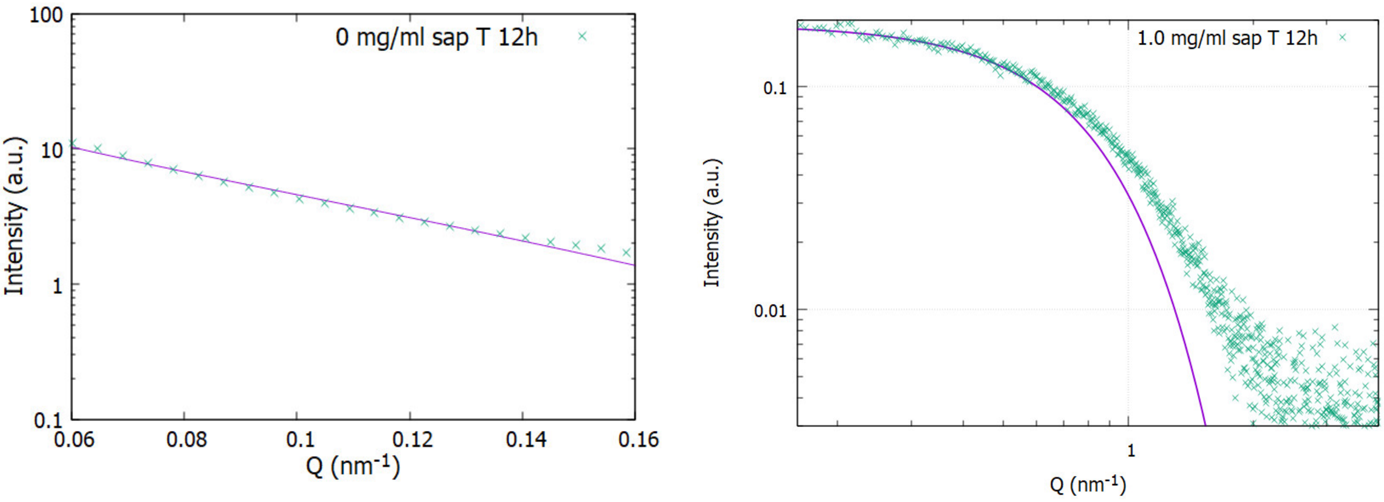

Figure S4. Experimental SAXS curves of human insulin at the end of the kinetics of aggregation. Left panel reports SAXS experimental curve of the final state of human insulin aggregation, in absence of saponins. Right panel reports SAXS experimental curves of human insulin at the end of the kinetics, in presence of saponins. Continuous purple lines represent the theoretical fitting obtained by Guinier rod-like (for the sample without saponins) and by Guinier (for the sample with saponins) approximations.

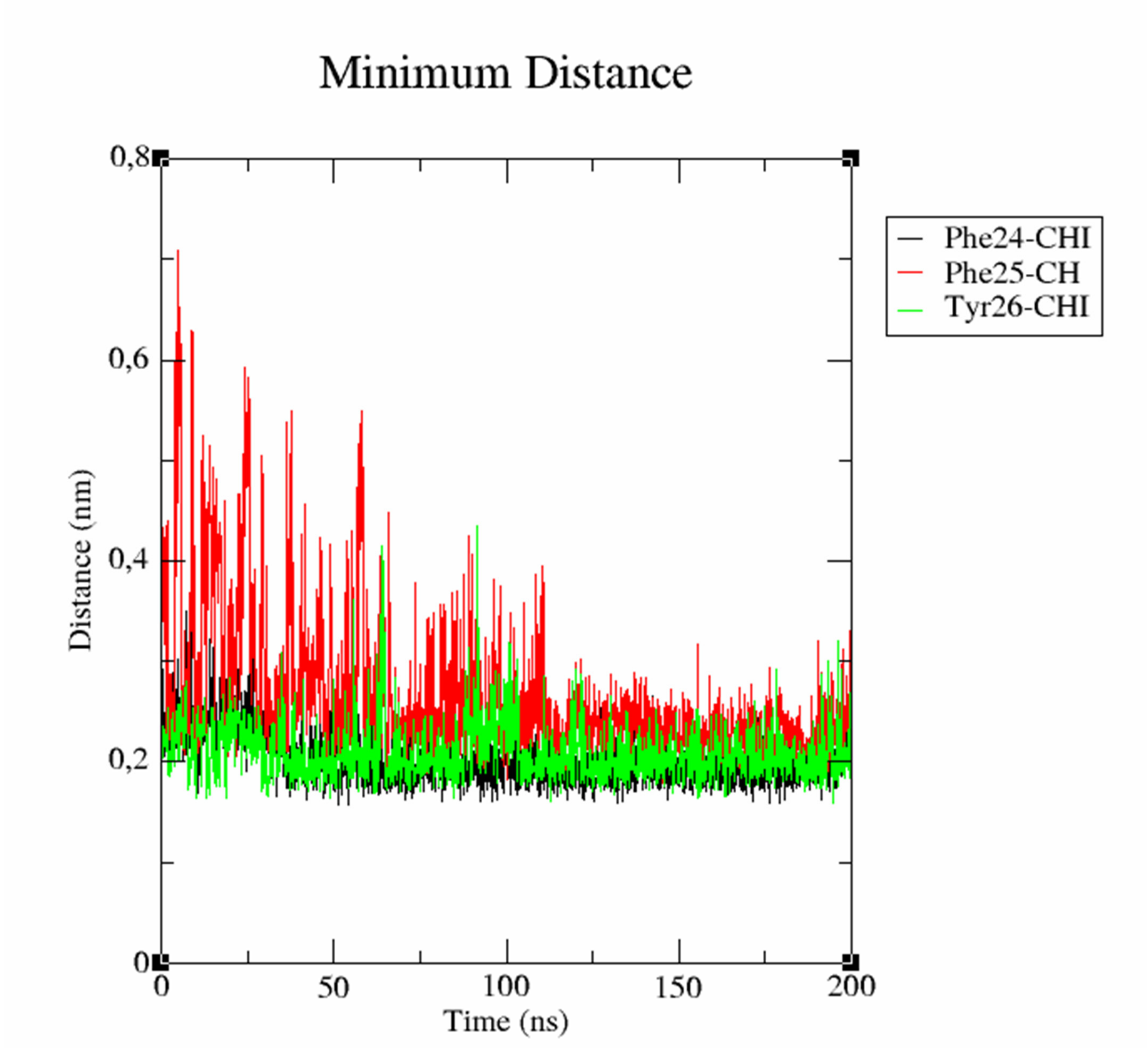

Figure S5. Intermolecular distances between the four Chichigenin B molecules and Phe24, Phe25 and Tyr 26 of insulin along the 200 ns MD simulation.

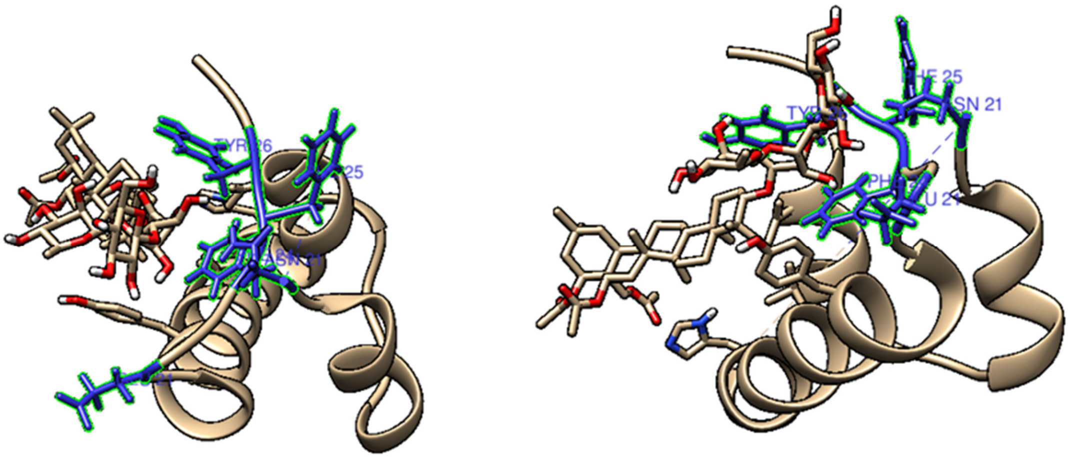

Figure S6. 1:1 Chichigenin B / insulin – left top view, right side view. Steady state structure after 100 ns MD simulation in water solution.

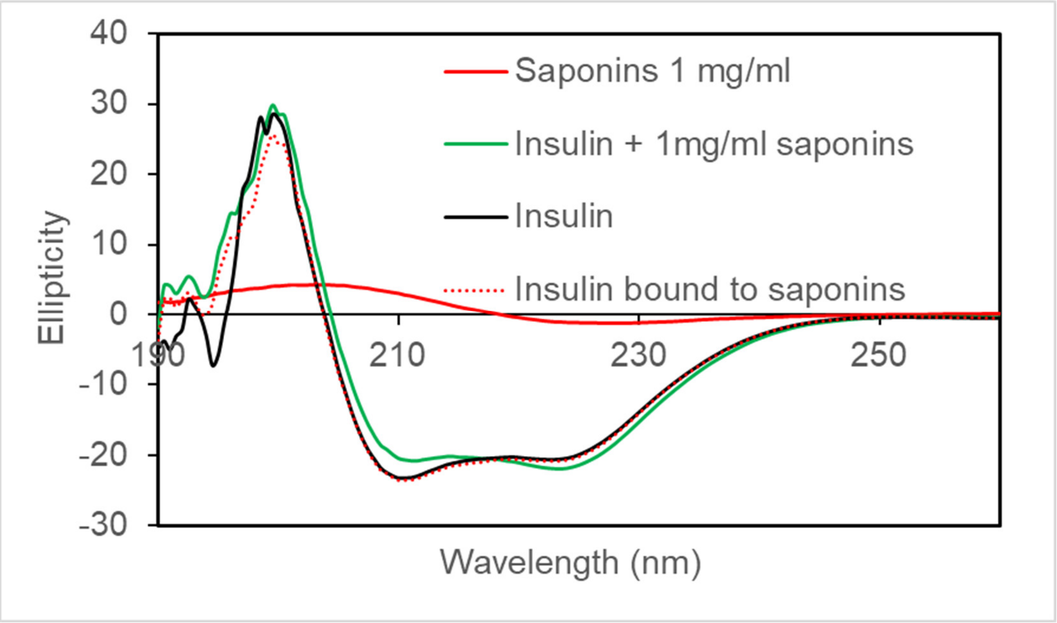

Figure S7. CD spectra of human insulin in presence and in absence of 1 mg/ml saponins, together with saponins solution, as in the legend. Insulin CD spectrum compared with the one obtained from the difference between the insulin-saponins sample and the saponins-alone sample, attributed to the structure of insulin bound to saponins, are very similar.
